# Poster Session I - A115 GAMING IN GASTROENTEROLOGY: A RANDOMIZED CONTROLLED TRIAL

**DOI:** 10.1093/jcag/gwaf042.115

**Published:** 2026-02-13

**Authors:** J E Perron, M J Coffey, A Doja, C Y Ooi

**Affiliations:** University of Ottawa, Ottawa, ON, Canada; University of New South Wales Medicine & Health, Sydney, New South Wales, Australia; University of Ottawa, Ottawa, ON, Canada; University of New South Wales Medicine & Health, Sydney, New South Wales, Australia

## Abstract

**Background:**

Serious games (SGs) can augment medical education through technology-enhanced simulation. PlayMed (PM), a highly immersive and experiential SG, was designed to teach the clinical assessment and management of pediatric acute gastroenteritis (AG) and acute pancreatitis (AP).

**Aims:**

To determine if the use of PM leads to superior outcomes based on the Kirkpatrick Model of Evaluation (KP) by assessing learner reaction (KP 1), knowledge acquisition (KP 2), and self-perceived behaviour (KP 3) compared to the use of written clinical guidelines (GL).

**Methods:**

This is a randomized controlled trial among Pediatric residents at the Children’s Hospital of Eastern Ontario. Participants were block-randomized to the PM or GL group for one week and then completed a Likert-style questionnaire and 20 multiple choice questions (MCQs).

**Results:**

Twenty participants were randomized; 19 completed the assessment and were included in the analysis (PM n = 9, GL n = 10). No significant differences in baseline age or sex between groups. For KP 1, PM participants found the activity significantly more engaging (78% vs. 20%, p = 0.02) and enjoyed the design and elements (78% vs. 0%, p < 0.001) compared to the GL group. Although not statistically significant, more PM participants enjoyed the activity overall (44% vs. 10%) and enjoyed the format (56% vs. 40%). The GL group found the activity easy to use (100% vs. 22%, p < 0.001). For KP 2, the GL group reported improved understanding of the topics (100% vs. 44%, p = 0.01). However, there was no significant difference in MCQ scores between groups. For KP 3, the GL group reported improved confidence in managing AG (80% vs. 22%, p = 0.02), but no significant difference for AP. In the PM subset analysis, higher computer proficiency was significantly associated with improved self-perceived overall understanding (p = 0.04). Additionally, preference for simulation-based learning was significantly associated with enjoying PM and perceiving it as a practical and engaging way to develop clinical skills (p = 0.04 for both comparisons).

**Conclusions:**

Overall, PM was more engaging and enjoyable compared to GL. In the PM group, preference for simulation-based learning was associated with enjoyment and self-perceived development of clinical skills. GL were reported as easier to use and demonstrated more confidence in managing AG.

**Funding Agencies:**

None

